# A review of reproducible and transparent research practices in urology publications from 2014 to2018

**DOI:** 10.1186/s12894-022-01059-8

**Published:** 2022-07-11

**Authors:** Shelby Rauh, Bradley S. Johnson, Aaron Bowers, Daniel Tritz, Benjamin Matthew Vassar

**Affiliations:** Oklahoma State University Center for Health Sciences, 1111 W 17th St., Tulsa, OK 74107 USA

**Keywords:** Reproducibility, Replicability, Transparency, Urology, Urologic research

## Abstract

**Background:**

Reproducibility is essential for the integrity of scientific research. Reproducibility is measured by the ability of different investigators to replicate the outcomes of an original publication using the same materials and procedures. Unfortunately, reproducibility is not currently a standard being met by most scientific research.

**Methods:**

For this review, we sampled 300 publications in the field of urology to assess for 14 indicators of reproducibility including material availability, raw data availability, analysis script availability, pre-registration information, links to protocols, and if the publication was available free to the public. Publications were also assessed for statements about conflicts of interest and funding sources.

**Results:**

Of the 300 sample publications, 171 contained empirical data available for analysis of reproducibility. Of the 171 articles with empirical data to analyze, 0.58% provided links to protocols, 4.09% provided access to raw data, 3.09% provided access to materials, and 4.68% were pre-registered. None of the studies provided analysis scripts. Our review is cross-sectional in nature, including only PubMed indexed journals-published in English-and within a finite time period. Thus, our results should be interpreted in light of these considerations.

**Conclusion:**

Current urology research does not consistently provide the components needed to reproduce original studies. Collaborative efforts from investigators and journal editors are needed to improve research quality while minimizing waste and patient risk.

**Supplementary Information:**

The online version contains supplementary material available at 10.1186/s12894-022-01059-8.

## Background

Reproducibility—determined by the availability of materials, raw data, analysis procedures, and protocols used to conduct original research so that it may be reproduced by others is crucial to establishing credible and reliable research that ultimately governs clinical practice. Recent evidence suggests that up to 90% of preclinical research may not be reproducible [1]. A recent survey of over 1,500 researchers concurred with this assessment, with the vast majority believing that biomedical research is experiencing a “reproducibility crisis” [2]. Several explanations have been suggested for why reproducibility has become an issue, with pressure to publish and the race to be the first to report new findings being among the most likely causes [3]. When research is not reproducible, time and money are wasted reproducing erroneous results, and patients may be exposed to ineffective or harmful therapies [4].

Concerns about reproducibility span from preclinical to clinical research. Consider prostate cancer research as an example. On the diagnostic side, in vitro studies are performed on prostate biopsy samples to advance our understanding of early detection and diagnosis. Widespread misuse of immunohistochemical staining contributes to the lack of research reproducibility. Sfanos et al. argued that ubiquitously used research-grade antibodies within the biomedical research community (as opposed to clinical grade used for patient diagnosis) are not routinely validated in investigators’ labs, which may lead to varying results that cannot be reproduced in subsequent studies [5]. On the other end of the research spectrum, randomized clinical trials are conducted to evaluate the efficacy of new therapeutic agents for the prevention or treatment of prostate cancer. In one large-scale randomized trial, Thompson et al. compared the effects of finasteride against placebo for prostate cancer prevention. These investigators found that finasteride prevented or delayed the development of prostate cancer but also led to an increased risk of higher-grade cancer upon detection [6]. The raw data from this clinical trial were not made entirely available because of patient privacy and data “messiness.” Some investigators have attempted to re-analyze the trial data but the results have been mixed [7, 8]. Since then, Baker et al. proposed a method to overcome issues of privacy and messiness,while at the same time fostering the reproducibility of trial outcomes [9].

Thus, when a study does not report the components needed for reproducibility or when studies are not replicated by other researchers, it is difficult to determine the credibility of the original findings. Our study examines existing research in urology and determines how often studies include markers of reproducibility and how frequently studies are replicated. We believe that our research will bring emphasis to the issue of reproducibility in urology research, where the topic has not been well-explored. The specific types of reproducibility evaluated were computational and emprical. Empirical reproducibility means that a publication has provided sufficient protocols and methodology to replicate the study design [10]. Computational reproducibility uses data sharing to recalculate and verify study outcomes.

## Methods

This is a methodological review of urology publications. We used the methodology by Hardwicke and colleagues [11] with modifications mentioned below. In an effort to foster transparency and reproducibility, we have uploaded our protocol, data extraction form, and other necessary materials for public viewing on the Open Science Framework (https://osf.io/n4yh5/).

### Clarifying definitions

Some confusion exists between the terms “reproducibility” and “replicability.” For the purposes of this investigation, we use the National Academies’ definitions: *reproducibility* is “obtaining consistent results using the same input data; computational steps, methods, and code; and conditions of analysis,” whereas replicability is “obtaining consistent results across studies aimed at answering the same scientific question, each of which has obtained its own data” [12].

### Journal selection

We used the National Library of Medicine (NLM) catalog to search for all relevant journals using the subject terms tag “Urology” [ST]. This search was performed on May 30, 2019. The inclusion criteria required that journals provided full-text publications in English and were MEDLINE-indexed. The list of journals in the NLM catalog fitting the inclusion criteria were then extracted using the electronic International Standard Serial Number (ISSN) or the linking ISSN when the electronic ISSN was unavailable. PubMed was searched with the list of ISSN to identify all publications within from January 1, 2014 to December 31, 2018 and a simple random sample of 300 publications exracted that met the inclusion criteria for our analysis using the RAND function in Microsoft Excel (https://osf.io/csf5t/). We chose random sampling so that each publication would have an equal opportunity for selection and to serve as an unbiased representation of the population of publications.

### Data extraction training

The two investigators responsible for data extraction (SR and BJ) underwent a full day of training to ensure adequate inter-rater reliability. The training included an in-person session that reviewed the project study design, protocol, data extraction form, and examples of where information may be contained using two example publications. The investigators were then given three example publications from which to extract data in a blinded fashion. Following data extraction, the pair reconciled differences between them. This training session was recorded from the presenter’s point of view (DT) and listed online for reference (https://osf.io/tf7nw/). As a final training exercise, investigators extracted data from the first 10 publications of their sample. The investigators then held a meeting to reconcile any differences in the data before extracting data from the remaining 290 publications.

### Data extraction

Data extraction on the remaining 290 publications was then conducted in a duplicate, blinded fashion. A final consensus meeting was held with both investigators to resolve disagreements. A third investigator (DT) was available for adjudication but was not needed. We extracted data using a pilot-tested Google form based on Hardwicke and colleagues but with modifications.This form contained information necessary for a study to be reproducible, such as the availability of materials, data, protocols, or analysis scripts (https://osf.io/3nfa5/). The data extracted varied based on the study design with studies having no empirical data being excluded (e.g., editorials, commentaries [without reanalysis], simulations, news, reviews, and poems). The form also included the five-year and most recent-year impact factors when available, and expanded the study design options to include cohort studies, case series, secondary analyses, chart reviews, and cross-sectional studies.We also expanded the funding options to include university, hospital, public, private/industry, non-profit, or mixed funding.

### Evaluation of open access status

We evaluated all 300 publications to determine whether they were freely available online through open access. We searched the Open Access Button (openaccessbutton.org) with publication titles and DOI numbers. This tool actively searches for the full-text online. If the Open Access Button was unable to find the publication, then SR and BJ searched Google Scholar and PubMed to determine if the full-text was available as open access on the journal website.

### Evaluation of replication and whether publications were included in research synthesis

For empirical studies, excluding meta-analysis and commentary with analysis, we searched the Web of Science to determine whether the publication was cited in a replication study, meta-analysis, or systematic review. To conduct this search, two authors (SR and BJ) first searched WoS for each included study. If the target study had been cited by another publication, then we analyzed those citing publication titles for terms to indicate that they were systematic reviews (i.e., “systematic review, Cochrane”), meta-analyses, or replication studies. The Web of Science additionally lists information important for our study, such as the country of journal publication, five-year impact factor (when available), and most recent impact factor.

### Statistical analysis

We report descriptive statistics for each of our findings using analysis functions within Microsoft Excel. The main findings are the number reported and the portion of total analyzed studies (Table 1).

## Results

### Included sample and characteristics

**Fig. 1:**
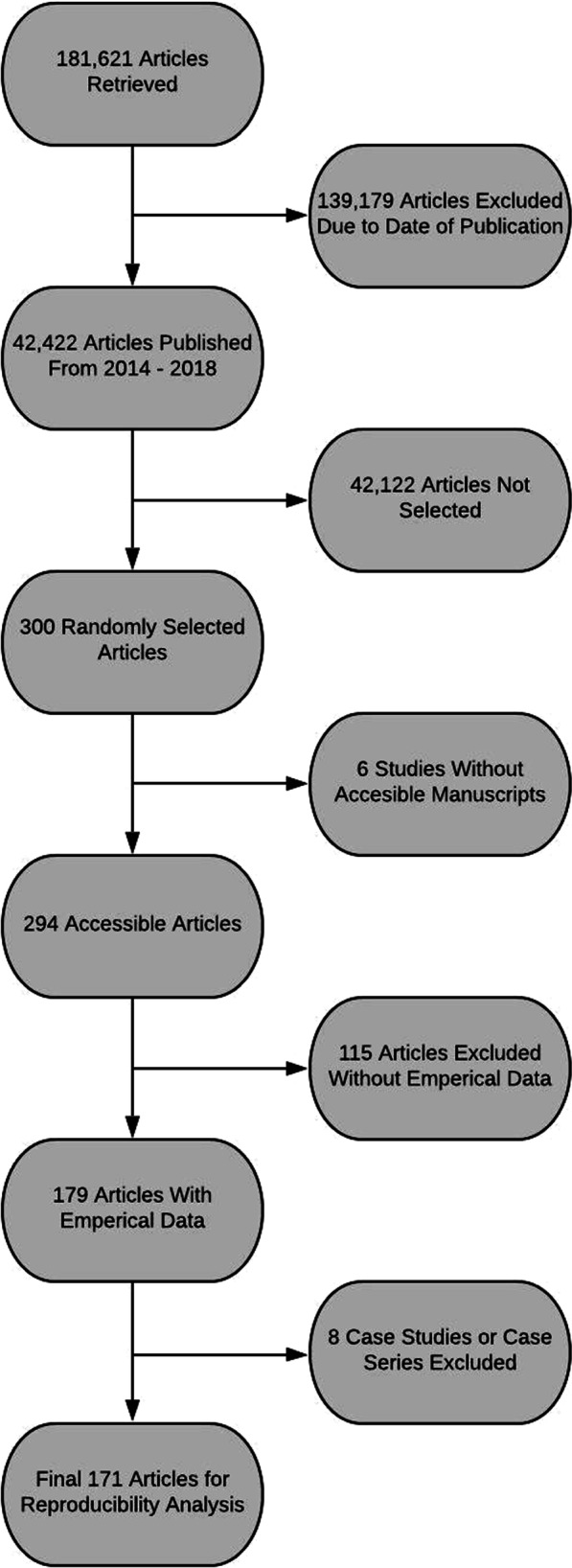
Flow diagram of included and excluded studies for the reproducibility analysis

Our inclusion criteria resulted in 42,422 articles from 46 urology journals found in the National Library of Medicine catalog. Of the articles resulted from the inclusion criteria, 300 articles were randomly chosen for analysis. Six articles were not analyzed due to lack of access to the manuscript. The remaining 294 articles were assessed to determine the five-year impact factor of their corresponding journals. Twenty of the 294 articles came from journals without five-year impact factors. Thus, journals of the 274 studies reported a median of 2.466 as their five-year impact factor with an interquartile range from 1.898 to 4.925. In addition, a full assessment of the original 300 articles revealed that 88 (29.33%) were openly accessible through Open Access Button or other means. Over half the included studies (55.44%, 163/294) provided a statement revealing that their study was without a conflict of interest. However, 32.31% (95/294) of the included studies did not provide any type of conflict of interest statement. Nearly two-thirds (62.93%, 185/294) did not state if or from where they received funding. As for the studies that provided a statement, most studies did not receive funding (31/294) while those that did receive funding did so through public entities (23/294). A PRISMA diagram detailing included and excluded studies can be found in Fig. 1. Other characteristics of our included studies can be found in Table 2 and Addtional file 1: Table S1.

**Table 1:** Types of Characteristics Associated with Reproducibility. Sample sizes (*N*) depends on study type. Protocol for measured characteristics is found online: https://osf.io/x24n3/

Reproducibility markers		Importance of each marker in regard to transparency and reproducibility
*Accessibility*		
All (*N* = 300)	Article accessibility (Is the article available to the public without a paywall?)	Accessible research allows for a larger audience to assess and replicate a study’s findings
*Funding*		
Included studies (*N* = 294)	Funding statement (Do authors provide a statement to describe if or how the study was funded?)	Including a funding statement provides greater transparency to readers. This increased transparency reveals any signs of bias or influence in the study’s methodology
*Conflict of Interest*		
Included studies (*N* = 294)	Conflict of interest statement (Do the authors reveal any conflicts of interest in their manuscript?)	Conflict of interest statements give the authors a chance to be transparent about relationships with entities that may try to influence a study’s findings
*Publication citations*		
Empirical studies^a^ (*N* = 171)	Systematic review/meta-analysis citations (Has the study been cited by data synthesis study designs such as systematic reviews or meta-analyses?)	Systematic reviews and meta-analyses synthesize information in studies that may have been replicated. The synthesis of information reveals a more complete answer to the question being investigated
*Analysis scripts*		
Empirical studies^b^ (*N* = 171)	Availability statement (Is there a statement in the manuscript describing the accessibility of the analysis script?)	Having the analysis script allows raw data to be analyzed exactly as the authors did in the original study, allowing others to replicate the data analysis correctly
	Location of analysis script (Where can the analysis script be found? Supplementary materials?)	
	Accessibility (Can a reader access the analysis script through the manuscript online or through other methods?)	
*Materials*		
Empirical studies^c^ (*N* = 162)	Availability statement (Is there a statement in the manuscript describing the accessibility of additional materials to the study?)	Additional materials allows readers to learn what is needed to reproduce the study, enabling the study to be replicated
	Location of additional materials (Where can the additional material be found? Supplementary materials?)	
	Accessibility (Can a reader access additional material through the manuscript online or through other methods?)	
*Pre-registration*		
Empirical studies^b^ (*N* = 138)	Availability statement (Is there a statement in the manuscript describing whether the study was pre-registered or not?)	Pre-registering a study prevents any tampering of the study design throughout implementation of the study, increasing the reliability of the study. Pre-registration also can provide components that may aid in replicating a study
	Location of registration(Where was the study registered?)	
	Accessibility of the registration (Is the registration accessible?)	
	Components included in registration (What components of the study were found in the registration?)	
*Protocols*		
Empirical studies^b^ (*N* = 171)	Availability statement (Is there a statement in the manuscript describing whether the study protocol was available or not?)	Access to a detailed protocol allows others to know what, where, why, and how the study was performed, aiding others in the replication of the original study
	Components (What components of the study were found in the protocol?)	
*Raw data*		
Empirical studies^b^ (*N* = 171)	Availability statement (Is there a statement in the manuscript describing the accessibility of raw data from the study?)	Raw data provide insight into the author’s thoughts and actions throughout implementation of the study, aiding others in replication of the original study. Additionally, raw data provide transparency to what is presented in the study’s findings
	Method of availability (Where can the raw data be found? Supplementary materials?)	
	Accessibility (Can a reader access raw data through the manuscript online or through other methods?)	
	Components (Are all the components of raw data that is needed to replicate the study available?)	
	Clarity (Are the raw data understandable?)	

^a^Empirical studies contain empirical data, e.g., clinical trials, cohort studies, case series, case reports, case–control, secondary analysis, chart review, commentaries (with data analysis), laboratory, and cross-sectional designs

^b^Empirical studies that are case reports, case series, or studies without World of Science access were excluded from the reproducibility analysis (i.e., materials, data, protocol, and registration were excluded) as recommended by Hardwick et al. [10]

^c^Empirical studies that are case reports, case series, commentaries with analysis, meta-analyses, or systematic reviews were excluded as they are not expected to provide additional materials

**Table 2:** Characteristics of Included Publications

Characteristics		Variables
Characteristics of included publications		
		*N* (%)
Funding (*N* = 294)	University	4 (1.36%)
	Hospital	1 (0.34%)
	Public	23 (7.82%)
	Private/Industry	20 (6.80%)
	Non-profit	2 (0.68%)
	Mixed	28 (9.52%)
	No statement listed	185 (62.93%)
	No funding received	31 (10.54%)
Type of study (*N* = 294)	No empirical data	115 (39.12%)
	Meta-analysis	9 (3.06%)
	Chart review	10 (0.34%)
	Clinical trial	22 (7.48%)
	Case study	6 (2.04%)
	Case series	2 (0.68%)
	Cohort	94 (31.97%)
	Case sontrol	2 (0.68%)
	Survey	8 (2.72%)
	Laboratory	17 (5.78%)
	Other	9 (3.06%)
5-year impact factor (*N* = 274)	Median	2.466
	1st Quartile	1.898
	3rd Quartile	4.925
	Interquartile range	1.898–4.925

### Characteristics associated with reproducibility

The only studies that were assessed for reproducibility were those with empirical data. Thus, the 115 articles that did not contain empirical data were excluded from the initial 294 studies. In addition, we removed a total of eight case studies and case series due to the inability of these study types to be reproduced. The final number of studies assessed for reproducibility was 171. Of the final number of studies, 95.32% (163/171) did not provide a pre-registration statement. Among the eight studies that provided a pre-registration statement, four had accessible links to the pre-registration. Nearly all of the analyzed studies did not provide a data availability statement (94.74%, 162/171). None of the seven studies that claimed data was available provided enough raw data for the study to be reproduced. Similarly, 96.30% (156/162) of our analyzed studies did not provide a material availability statement.Six studies did provide a material availability statement; of these six studies, five stated that materials were available, but only four studies provided working links to the materials. Only one of the 171 studies provided a full protocol in their manuscript. None of the 171 studies in our assessment provided an analysis script availability statement. More characteristics associated with reproducibility can be found in Addtional file 1: Table S1 and Addtional file 2: Table S2.

## Discussion

Our review revealed concerning findings regarding the reproducibility of research in urology literature. As types of reproducibility rely on different components of the manuscript and study design, they are identified independently of each other. A study may provide information to be replicated but not the data to reproduce the calculations; thus multiple components were evaluated and reported. Only nine studies made statements regarding the availability of data with only seven of those actually making their data available. Fewer than half the studies in our sample were available through the Open Access Button and detailed protocols and pre-registration were rare. There was one trial in our sample that claimed to be a replication of a previous study and even this manuscript failed to include any of the markers of reproducibility that we assessed. These findings are similar to what Hardwicke et al. found in a survey of reproducibility in social sciences literature [11].

Our study revealed that only one study contained a link to protocols while no studies provided analysis scripts and only six provided materials statements. These are three of the most important elements in reproducing a study. Protocols provide details about how each step of the study was performed andto an extent much deeper than would be relevant to the average person reading the methods Sect. [13, 14] Similarly, analysis scripts are crucial for recreating the original analysis in a stepwise manner [15]. Materials include items necessary for the study to be performed, including forms, questionnaires, devices, software programs, and more [16]. Some investigators have posited that freely providing these elements invites plagiarism of study design, a major concern for researchers with limited time and funding, and with pressure on them to publish [17]. However, it can also be argued that the failure to adequately report study methodologies is even more detrimental. Consider the 1989 incident in which Stanley Pons and Martin Fleischmann announced the development of a method for producing nuclear fusion at room temperatures. These scientists bypassed peer review and reported results directly to the public to protect their claims to priority and intellectual property. Scientists from across the globe attempted, albeit unsuccessfully, to reproduce these results using the ill-reported methodology described in the press release. These attempts led to wasted time and resources and marred cold fusion research for years to come [18]. Chan et al. suggested placing protocols in a lockbox and making them available upon data release to protect intellectual property while maintaining reproducible research [19]. At the very least, authors should make a statement in their manuscript that these crucial elements of reproducibility are available upon reasonable request.

Pre-registration is one of the best ways to increase transparency and reproducibility in research, yet only eight studies from our sample were pre-registered. Pre-registration of trials encourages transparency in research by outlining the intended outcomes, interventions, protocols, and methods of analysis before the study is actually conducted [20]. When trials are not pre-registered, investigators have the freedom to manipulate data to obtain significance (P-hacking) [21], hypothesize after results are known (HARKing) [22], switch primary outcomes [23], or deviate from a priori protocols [24]. Several researchers, including Nosek et al. have called for widespread adoption of pre-registration, citing its value in increasing transparency, rigor, and reproducibility [25]. Early results of pre-registration are positive, with pre-registered studies showing a significant increase in null findings [26]. The Open Science Framework (OSF) hosts pre-registration free of charge and also provides pre-registration templates and instructional guides [27, 28]. The questionable research practices (QPRs) that can be mitigated with pre-registration are not always intentional. Authors are not always aware of information to include in the manuscript to increase reproducibility and transparency. Journals would do well to require pre-registration for any study to be considered for publication, especially those wishing to publish in journals with high impact factors.

Data availability is another area where urology research falls short. Some journals, including *European Urology*, have begun to require authors to describe in their manuscript how readers can access underlying data while other journals mandate the inclusion of study protocols, analysis scripts, and any other items a researcher would need to replicate the original study [29, 30]. Beginning in 2019, the ICMJE mandated data sharing by all prospective clinical trials submitted for publication to an ICMJE member journal [31]. Showing that such policies can be successful, *PLOS One*, another journal requiring data availability, reported that 20% of their studies hosted their data on a third-party website, 60% provided their data in a supplement and the remaining 20% made their data available upon reasonable request [32]. These initiatives are steps in the right direction and we propose a few more possibilities that could improve reproducibility in urology research.

The Repeat framework was designed by McIntosh et al. to improve reproducibility in research. It is an easy-to-use checklist and can be adapted for most studies. The check-list includes 119 unique variables that aim to improve study quality in areas such as research design, data collection methods, data management, data analysis, and documentation [10]. Additionally, the OSF developed the Transparency and Openness Promotion (TOP) Guidelines. The TOP guidelines provide eight modular standards designed to increase transparency, disclosure, openness, and collaboration [33]. The EQUATOR network aims to improve research reporting and manuscript writing through the use of reporting guidelines [34, 35]. These guidelines, available for nearly every type of study imaginable, ensure that manuscripts are written in a transparent way, encouraging reproducibility and accurate reporting of findings [36]. Some journals have begun to require the use of reporting guidelines in the studies they publish [37–39].

Ninety-five studies in our sample failed to provide a conflict of interest statement, a concerning finding given the amount of conversation about conflicts of interest in urology recently [40, 41]. A recent review by Jimbo et al. found poor conflict of interest disclosure rates among robotic pediatric urological surgery studies, with 80.4% of authors reporting payments from Intuitive Surgical, the maker of the da Vinci surgical system [42]. Additionally, they found that studies with first or last authors with a history of payments were more likely to endorse robotic surgery [42]. Most journals require some form of conflict of interest disclosure, yet undisclosed conflicts continue to be a problem [43, 44]. Clearly, self-disclosure is not a viable solution, and it may take additional journal staff or third parties to investigate the authors of every submitted article to get anywhere near the desired rate of 100% disclosure.

Our study has both strengths and limitations. Regarding strengths, we applied double data extraction procedures, which is considered a best practice methodology by the systematic review community and is recommended in the *Cochrane Handbook for Systematic Reviews of Interventions* [45]. To foster study reproducibility and transparency, we have made all relevant study materials available publicly on Open Science Framework. Concerning limitations, our review is cross-sectional in nature, including only PubMed-indexed journals published in English, and within a finite time period. Thus, our results should be interpreted in light of these considerations. Additionally, many replication studies are not published. In fact, many replication studies are never submitted for publication [2]. In recent years, some organizations, including Elsevier, have taken steps to encourage the submission and publication of replication studies; however, we are not yet at a point where they are common in biomedical literature [46]. Further, as we only analyzed publications in English and located within the NLM, we cannot speak to the generalization of our findings to publications that fall outside that scope. We did not attempt to contact authors for data availability, analysis scripts, protocols, or any other markers of reproducibility. This decision was made due to the large number of publications that we analyzed as well as a common research phenomenon wherin, low response rates and limited cooperation often cause research to become stagnant [47, 48]. This decision limits the generalizability of our findings only to publications that openly publish all their data. Finally, there are other forms of reproducibility that were not evaluated in this publication such as statistical, inferential, or study sampling which could be areas of future research.

## Conclusions

Current urology research does not consistently provide the components needed to reproduce original studies. Collaborative efforts from investigators and journal editors are needed to improve research quality while minimizing waste and patient risk.

## Supplementary Information

**Additional file 1**: Table S1. Additional Characteristics of Reproducibility in Urology Studies I.

**Additional file 2**: Table S2. Additional Characteristics of Reproducibility in Urology Studies II.

## Data Availability

Dara are available in a public, open access repository. Data and study materials are available through the Open Access Framework Database (https://osf.io/n4yh5/).
