# Supplementary material for: A review of reproducible and transparent research practices in urology publications from 2014 to2018
Source: BMC Urol. 2022 Jul 11;22:102. doi: 10.1186/s12894-022-01059-8 (PMC9277815; doi:10.1186/s12894-022-01059-8)
Supplement: Supplementary file 1 — Additional file 1: Table S1. Additional Characteristics of Reproducibility in Urology Studies I. [file 12894_2022_1059_MOESM1_ESM.docx]

**Supplemental Table 1: Additional Characteristics of Reproducibility in Urology Studies I**

| **Additional Characteristics of Reproducibility in Urology Studies** | | |
| --- | --- | --- |
| **Characteristic** | | **Variables** |
|  | | ***N* (%)** |
| **Conflict of interest statement (*N*=294)** | Statement, one or more conflicts of interest | 36 (12.24%) |
|  | Statement. no conflict of interest | 163 (55.44%) |
|  | No conflict of interest statement | 95 (32.31%) |
| **Data availability (*N*=171)** | Statement, some data are available | 7 (4.09%) |
|  | Statement, data are not available | 2 (1.17%) |
|  | No data availability statement | 162 (94.74%) |
| **Material availability (*N*=162)** | Statement, some materials are available | 5 (3.09%) |
|  | Statement, materials are not available | 1 (0.62%) |
|  | No materials availability statement | 156 (96.30%) |
| **Protocol availability (*N*=171)** | Full protocol | 1 (0.58%) |
|  | No protocol | 170 (99.42%) |
| **Analysis scripts (*N*=171)** | Statement, some analysis scripts are available | 0 (0%) |
|  | Statement, analysis scripts are not available | 0 (0%) |
|  | No analysis script availability statement | 171 (100%) |
| **Replication studies**  **(*N*=171)** | Novel study | 170 (99.42%) |
|  | Replication | 1 (0.58%) |
| **Open Access (*N*=300)** | Yes ­ found via Open Access Button | 87 (29.00%) |
|  | Yes ­ found article via other means | 1 (0.33%) |
|  | Could not access through paywall | 212 (70.67%) |
| **Cited in Systematic review/**  **Meta-analysis (a) (*N*=169)** | No citations | 140 (82.84%) |
|  | Single citation | 20 (11.83%) |
|  | 1–5 itations | 9 (5.33%) |
| Abbreviations: CI, Confidence Interval; (a), No studies were explicitly excluded from the systematic reviews or meta-analyses that cited the original article. | | |
|  |  |  |
|  |  |  |
| **Pre-registration (*N*=171)** | Statement, says was pre-­registered | 8 (4.68%) |
|  | Statement, says was not pre-registered | 0 |
|  | No, there is no pre-registration statement | 163 (95.32%) |
| **Test subjects (*N*=294)** | Animals | 11 (3.74%) |
|  | Humans | 204 (69.39%) |
|  | Neither | 79 (26.87%) |
| **Country of journal publication (*N*=294)** | US | 182 (61.90%) |
|  | UK | 34 (11.56%) |
|  | Germany | 2 (0.68%) |
|  | India | 11 (3.74%) |
|  | Italy | 3 (1.02%) |
|  | Unclear | 9 (3.06%) |
|  | Other (a) | 53 (18.03) |
| **Country of corresponding author**  **(*N*=294)** | US | 116 (39.46%) |
|  | China | 20 (6.80%) |
|  | UK | 14 (4.76%) |
|  | Germany | 10 (3.40%) |
|  | Japan | 13 (4.42%) |
|  | France | 6 (2.04%) |
|  | Canada | 14 (4.76%) |
|  | Italy | 24 (8.16%) |
|  | India | 2 (0.68%) |
|  | Spain | 5 (1.70%) |
|  | Unclear | 11 (3.74%) |
|  | Other | 59 (17.69%) |
