# Supplementary material for: A review of reproducible and transparent research practices in urology publications from 2014 to2018
Source: BMC Urol. 2022 Jul 11;22:102. doi: 10.1186/s12894-022-01059-8 (PMC9277815; doi:10.1186/s12894-022-01059-8)
Supplement: Supplementary file 2 — Additional file 2: Table S2. Additional Characteristics of Reproducibility in Urology Studies II. [file 12894_2022_1059_MOESM2_ESM.docx]

**Supplemental Table 2: Additional Characteristics of Reproducibility in Urology Studies II**

| **Additional Characteristics of Reproducibility in Urology Studies** | | |
| --- | --- | --- |
| **Characteristics** | | **Variables** |
|  | | ***N* (%)** |
| **Material availability (*N*=162)** | Personal or institutional | 0 |
|  | Supplementary information hosted by the journal | 5 |
|  | Online third-party | 0 |
|  | Upon request | 0 |
|  | Yes, material was accessible | 4 |
|  | No, material was not accessible | 1 |
| **Data availability (*N*=171)** | Personal or institutional | 0 |
|  | Supplementary journal information | 7 |
|  | Online third-party | 0 |
|  | Upon request | 0 |
|  | Other (b) | 0 |
|  | Yes, data could be accessed and downloaded | 3 |
|  | No, data could not be accessed and downloaded | 4 |
|  | Yes, data files were clearly documented | 2 |
|  | No, data files were not clearly documented | 1 |
|  | Yes, data files contain all raw data | 0 |
|  | No, data files do not contain all raw data | 3 |
|  | Unclear if all raw data was available | 0 |
| **Pre-registration (*N*=171)** | Pre-registered on ClinicalTrials.gov | 1 |
|  | Other (c) | 7 |
|  | Yes, pre-registration was accessible | 4 |
|  | No, pre-registration was not  accessible | 4 |
|  | Hypothesis was pre-registered | 2 |
|  | Methods were pre-registered | 1 |
|  | Analysis plan was pre-registered | 0 |
|  |  |  |
| **Protocol (*N*=171)** | Hypothesis was included in the protocol | 0 |
|  | Methods were included in the protocol | 0 |
|  | Analysis plan was included in the protocol | 0 |
